# Screening and Quality Evaluation of Submerged Culture Media Formulations for *Pleurotus pulmonarius*

**DOI:** 10.3390/jof12050310

**Published:** 2026-04-23

**Authors:** Jiling Song, Qiangjun Lang, Xingyu Lin, Song Wang, Weidong Yuan

**Affiliations:** 1 Hangzhou Academy of Agricultural Sciences, Hangzhou 310024, China; songjiling860605@163.com; 2 Dingxin Agricultural Technology Co., Ltd., Hangzhou 311301, China; 13868002095@163.com (Q.L.); lin1905615038@foxmail.com (X.L.); 18758215152@163.com (S.W.)

**Keywords:** *Pleurotus pulmonarius*, liquid spawn, medium screening, growth rhythm, quality standard, industrial production

## Abstract

The transition toward industrial-scale, year-round production of *Pleurotus pulmonarius* necessitates efficient and standardized spawn production. Liquid spawn technology plays a pivotal role in this process; however, recommended formulations and science-based quality criteria remain lacking. This study aimed to screen a high-performance liquid spawn medium and define key quality parameters for industrial application. Ten culture media formulations were evaluated to determine their effects on mycelial growth, as well as the subsequent yield and quality of fruiting bodies. The optimal formulation (Formula 4) contained glucose (1.6%), soybean meal (0.3%), corn flour (0.2%), peptone (0.2%), KH_2_PO_4_ (0.1%), and MgSO_4_ (0.055%). The growth rhythm of the selected formulation was meticulously tracked, leading to the identification of a critical inoculation window between 4.75 and 5.5 days. Spawn within this window exhibited a mycelial biomass of 1.60~1.86 g/L, pellet diameter of 1.83~1.92 mm, pellet density of 12.25~13.75 per mL, and fermentation broth pH of 6.35~6.44, which were strongly correlated with peak yield (up to 284 g/bag) and premium-grade ratio (up to 87.97%). The multi-parameter composite standard is proposed as a practical tool for quality control in industrial fermenters, enabling precise harvest timing and ensuring the consistent, high-yield, and high-quality production of *P. pulmonarius*.

## 1. Introduction

The *Pleurotus pulmonarius* mushroom is prized for its crisp texture, nutritional value, and short production cycle, making it particularly popular in Asia [1,2]. In recent years, sustained growth in market demand and rising labour costs have driven a profound transformation in the production models of the *P. pulmonarius* industry. This transformation involves a shift from the traditional, labour-intensive, and highly seasonal large-scale production to environmentally controlled, year-round, and highly efficient factory-based production [3]. This transition delivers significant economies of scale and improved supply stability, representing an inevitable pathway towards industrial modernization and enhanced international competitiveness [4].

However, such opportunities are accompanied by challenges. The key to factory-scale production lies in achieving standardization, precision and efficiency at every stage. As the ‘seed’ of edible mushroom cultivation, the quality and preparation efficiency of the fungal strain directly influence the success rate, cultivation cycle and final yield of subsequent cultivation [5]. Although traditional solid-state inoculation technology is well developed, it has several disadvantages, including prolonged cultivation cycles (typically 30~40 days), low inoculation efficiency, inconsistent Mycelial age and high risk of contamination. These factors significantly hinder the efficient, year-round industrial cultivation of *P. pulmonariu* [6]. Submerged culture technology is a highly promising solution to this problem [7]. Submerged culture offers several advantages, including rapid Mycelial growth (reducing the cultivation period to 5~7 days), consistent Mycelial age, uniform and convenient inoculation, and ease of large-scale propagation. These characteristics significantly shorten the cultivation cycles, increase the success rate of spawn bags, enhance equipment utilization, and reduce overall production costs [8,9]. This has been demonstrated in the industrialised production practices of edible fungi, such as *Flammulina velutipes* [10].

Considerable progress has been made in domestic research on the industrial production of *P. pulmonariu*. Valuable experience has been accumulated in areas such as facility environmental control [11], optimization of cultivation substrates [12] and management of fruiting bodies [13]. With regard to submerged cultures, existing studies have primarily focused on screening and comparing different strains, or using single-factor and response surface methods to optimize the carbon and nitrogen sources in culture media, aiming to maximize mycelial biomass [14]. One study established an optimal formulation consisting mainly of glucose and peptone [14], while another optimised the medium for wild oyster mushroom strains using corn flour and soybean meal [15]. These studies provide valuable references for preparing liquid spawn. However, systematic research on liquid spawn technology remains relatively scarce. Specifically, few studies have correlated physicochemical management indicators during spawn preparation, such as culture pellet morphology, density, and dynamic changes in fermentation broth pH, with final yield and quality. There is also a lack of studies that establish quantifiable quality standards applicable to production guidance [16]. Most existing studies only conduct simple comparisons among different cultivation conditions [17], formulations [18], or liquid [19] versus solid spawn cultures [8]. They lack a detailed analysis of the growth rhythm of liquid spawn cultures themselves and fail to integrate multiple parameters to establish a quality control system that can predict cultivation performance. Consequently, determining the optimal inoculation timing in industrial production still relies heavily on experience, which limits the full potential of the liquid spawn culture.

The aim of this study is to systematically investigate the cultivation techniques for liquid spawn of *P. pulmonarius*. Ten liquid medium formulations with different nutritional components were compared and evaluated to screen an optimal formulation suitable for producing high-quality, high-yield fruiting bodies. Based on the formulation, this study overcomes the limitation of focusing solely on final biomass, and precisely determines the dynamic variation curves of mycelial biomass, mycelial pellet morphology (diameter and density), fermentation broth pH, and mycelial germination rate throughout the entire fermentation process, thereby revealing the growth rhythm in depth. Finally, through comprehensive analysis and validation of the cultivation method, the optimal inoculation time window for liquid spawn was identified. A set of quantifiable and monitorable quality evaluation criteria for liquid spawn was established, with mycelial pellet morphology, biomass, and fermentation broth pH as core indicators, which are closely correlated with fruiting body yield. The results provide crucial theoretical foundations and technical parameters for the standardized, precise preparation of liquid spawn for the industrial production of *P. pulmonarius*, and effectively promote the industrial upgrading of its cultivation.

## 2. Materials and Methods

### 2.1. Test Strains

The *P. pulmonarius* strain ‘Jinxiu’ is the dominant cultivar used for industrial production in Zhejiang Province. This strain is preserved at the Edible Fungi Germplasm Resource Bank at the Hangzhou Academy of Agricultural Sciences.

### 2.2. Reagents and Instruments

Glucose, peptone, KH_2_PO_4_ and MgSO_4_ (all from Sinopharm Chemical Reagent Co., Ltd., Shanghai, China); an electronic balance (from Mettler Toledo, Greifensee, Switzerland); a constant-temperature shaking incubator (from Shanghai Zhichu Instrument Co., Ltd., Shanghai, China); a constant-temperature drying oven (from Shanghai Yiheng Scientific Instrument Co., Ltd., Shanghai, China); a pH meter (from Shanghai INESA Scientific Instrument Co., Ltd., Shanghai, China); a constant-temperature incubator (from Shanghai Yiheng Scientific Instrument Co., Ltd., Shanghai, China); an autoclave (from Shanghai Boxun Medical Biological Instrument Corp., Shanghai, China).

### 2.3. Screening of Submerged Culture Medium Formulations

The aim of this study was to identify suitable formulations of submerged culture medium for propagating the *P. pulmonarius* strain. Ten liquid media with different nutritional compositions were developed based on a widely used basic formulation by adjusting the inclusion and proportions of key components (soybean meal, peptone, Glucose, corn flour, KH_2_PO_4_ and MgSO_4_) (see Table 1 for details). Three 7 mm-diameter mycelial plugs were taken from the activated plate using a punch and inoculated into 250 mL conical flasks containing 150 mL of liquid medium. The flasks were then incubated in a shaking incubator at 22 °C with a rotation speed of 150 rpm. Subsequently, cultivation experiments were conducted to evaluate the fruiting performance of liquid spawn produced from each medium formulation, so as to determine the optimal medium.

### 2.4. Cultivation for Formulation Determination

To evaluate the effectiveness of different submerged culture formulations and cultivation durations in practical applications, *P. pulmonarius* fruiting body cultivation trials were conducted. The following indicators were measured:

Fruiting body yield: The first flush of *P. pulmonarius* fruiting bodies was harvested, their fresh weight was weighed using an electronic balance, and the yield per bag was recorded. Each treatment was replicated with 20 bags and the average value calculated.

High-quality mushroom rate: According to commercial grading standards for *P. pulmonarius*, the fruiting bodies that meet Grade A criteria were weighed separately (The pileus is dark grey, 3–5 cm in diameter; the Stipe is 3–6 cm long, approximately 1 cm in diameter, pure white, fleshy and free from deformities). Calculate their percentage of the total first flush yield. The formula for the high quality mushroom rate is:

High quality mushroom rate (%) = (Fresh weight of Grade A mushrooms/Fresh weight of total first flush yield) × 100%.

### 2.5. Documentation of Experimental Investigations

Systematic measurements were performed on the growth curve of the submerged culture using the formulation, so as to elucidate the dynamic growth patterns of *P. pulmonarius* submerged cultures. This laid a theoretical basis for industrial-scale production.

Samples were collected at 3, 3.5, 4, 4.25, 4.5, 4.75, 5, 5.25, 5.5, 5.75, 6, 6.25, 6.5, 6.75 and 7 days after incubation. The measured parameters included mycelial biomass, mycelial growth rate in the sawdust medium, mycelial pellet diameter and density, and fermentation broth pH. Each treatment was performed with six replicates. Through comprehensive analysis of the above parameters combined with the final fruiting performance, the key quality evaluation indicators for liquid spawn and their corresponding standard ranges were determined.

#### 2.5.1. Measurement of Mycelial Biomass

The fermentation broth to be tested was thoroughly shaken to ensure uniform dispersion. Mycelia were collected by filtering the broth through an 80-mesh sieve (aperture 0.18 mm). The collected mycelia were repeatedly rinsed with distilled water until no visible solid residues were observed in the filtrate. The washed mycelia were then transferred to a Petri dish with a predetermined weight. The Petri dish containing mycelia was dried in a constant-temperature drying oven at 60 °C until a constant weight was achieved, which was defined as a weight change of no more than 0.001 g between two consecutive weighings within a 12-h interval. After drying, the Petri dish was removed from the oven, cooled in a desiccator, and then weighed using an analytical balance. Each treatment was performed with six times. Mycelial biomass was calculated using the following formula:

Mycelial biomass (g/100 mL) = (Weight of dried Petri dish + Weight of mycelial) − Empty weight of Petri dish.

#### 2.5.2. Measurement of Pellet Diameter

The fermentation broth to be tested was thoroughly shaken. Then, 10 mL of the broth was pipetted into a Petri dish (9 cm in diameter), and the morphology of the mycelial pellets was observed under a microscope. Using the five-point sampling method, six morphologically intact mycelial pellets were randomly selected from the center and the four corners of the dish. Each mycelial pellet was photographed, and its diameter was measured. For each treatment, the diameters of 30 mycelial pellets were measured, and the average value was calculated. This average was taken as the result for that treatment.

#### 2.5.3. Determination of Mycelial Pellet Density

The fermentation broth to be tested was shaken thoroughly to ensure homogeneity. An accurate volume of 1 mL of the fungal suspension was pipetted and slowly poured into a 9 cm diameter Petri dish. The dish was placed on a level surface and allowed to stand briefly until the mycelial pellets had settled and stabilised. The total number of morphologically intact mycelial pellets was counted.

#### 2.5.4. Determination of Fermentation Broth pH

The fermentation broth to be tested was thoroughly shaken until homogeneous, and 5 mL was transferred into a sample cup. The pH meter was rinsed with deionized water, dried, and inserted into the fermentation broth. The pH value was recorded once the reading stabilised. Each sample was measured six times, and the average was calculated as the final pH value of the fermentation broth for that treatment.

#### 2.5.5. Mycelial Growth Rate

The mycelial growth rate on sawdust medium was determined to evaluate the germination and growth capacity of submerged cultures at different inoculation duration on solid substrates. Thirty grams of the medium were placed in a 9 cm diameter Petri dish and compacted to form a level surface. A sterile punch was used to create an inoculation hole (approximately 1 cm in diameter) at the center of the medium. A pipette was employed to accurately aspirate 0.5 mL of the submerged culture to be tested, which was then injected into the inoculation hole. The inoculated Petri dishes were placed in a constant-temperature incubator at 25 °C for static incubation. Six replicates were set for each treatment. During incubation, colony diameter was measured periodically using the cross-measurement method. The extension distance of mycelia on the medium surface, centred on the inoculation well, was recorded to calculate the average mycelial growth rate.

### 2.6. Cultivation Methods

According to the formula, the raw materials were mixed in the following proportions: 45% broadleaf hardwood sawdust, 25% cottonseed hulls, 10% corn cobs, 18% wheat bran and 2% lime. The moisture content was then adjusted to between 63% and 65%. Use a bagging machine to fill the bags. Each bag should be 18 cm in height and weighed 1.3–1.4 kg when wet. After autoclaving at 121 °C for 2.5 h and cooling, inoculate each bag with 25 mL of liquid spawn. Cultivation was carried out at 25 °C for 60 days until the spawn bags had matured. Standard fruiting body management was then implemented, and mushrooms were harvested when the pileus diameter reached 3–5 cm.

### 2.7. Data Processing

The experimental data were analyzed statistically using SPSS 22.0 software. One-way analysis of variance (ANOVA) was employed to test for significant differences between the treatments. Results are presented as the mean ± standard deviation (SD). Data charts were plotted using Origin 2021(9.8.0.200) software.

## 3. Results and Analysis

### 3.1. Screening Trial of Submerged Culture Formulations

**Figure 1:**
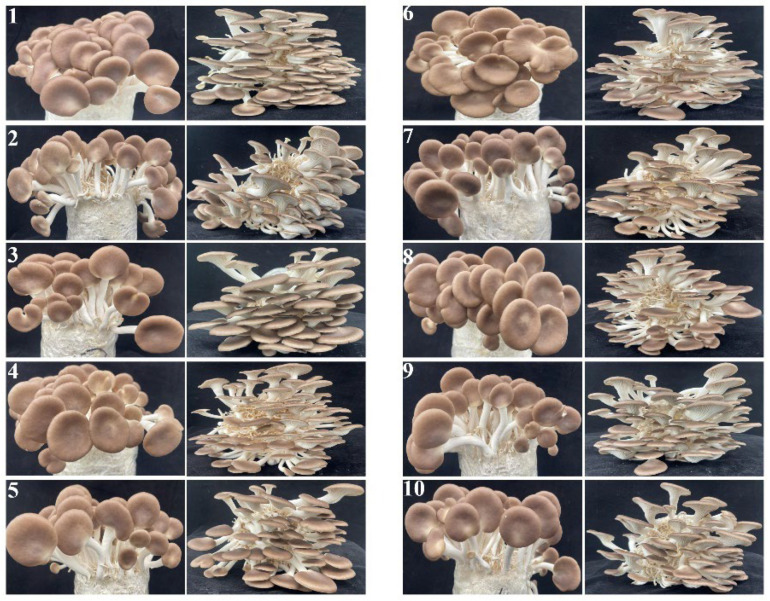
Mushroom fruiting performance of different submerged culture formulations. 1: formula 1; 2: formula 2; 3: formula 3; 4: formula 4; 5: formula 5; 6: formula 6; 7: formula 7; 8: formula 8; 9: formula 9; 10: formula 10.

A fruiting body cultivation trial was carried out using ten distinct liquid medium formulations for preparing liquid spawn of *P. pulmonarius*. The results are presented in Figure 1 and Table 2. Significant variations were observed among *P. pulmonarius* treatments in terms of yield, proportion of high-quality mushrooms and morphological traits of the fruiting bodies. Formulation 4 produced the highest yield (305.5 g/bag), which was significantly higher than that of the other treatments (*p* < 0.05), while formulation 10 had the lowest yield (176.5 g/bag). In terms of the high-quality mushroom ratio, formulation 8 performed the best, reaching 75.60%, whereas formulation 9 had the lowest value at 42.51%. Notably, formulation 4 also achieved a high-quality mushroom rate of 72.83%, which is relatively high. Morphometric analysis of the fruiting bodies indicated that formulation 4 had the longest pileus length (42.56 mm), the widest pileus (48.99 mm), and the largest stipe diameter (9.30 mm). Formulation 5 had the longest stipe length (45.21 mm), while formulation 1 had the thickest pileus (11.95 mm).

Taking all evaluation indicators into comprehensive consideration, formulation 4 showed the most excellent overall performance, with the highest yield of all the formulations, a high proportion of premium-quality mushrooms, and the most ideal morphology of the fruiting bodies (including pileus size and stipe thickness). Therefore, formulation 4 was identified as the optimal medium for the liquid propagation of the *P. pulmonarius* strain. Subsequent growth curve measurements and quality standard studies were conducted based on this formulation.

### 3.2. Fruiting Trials

**Figure 2:**
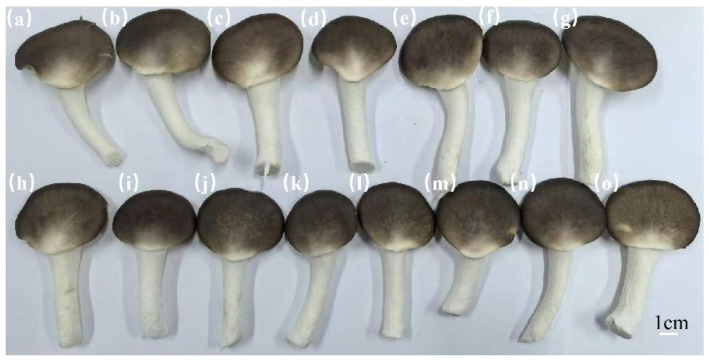
Fruiting body growth of submerged cultures at different inoculation durations. (**a**): 3 d; (**b**): 3.5 d; (**c**): 4 d; (**d**): 4.25 d; (**e**): 4.5 d; (**f**): 4.75 d; (**g**): 5 d; (**h**): 5.25 d; (**i**): 5.5 d; (**j**): 5.75 d; (**k**): 6 d; (**l**): 6.25 d; (**m**):6.5 d; (**n**):6.75 d and (**o**):7 d.

**Figure 3:**
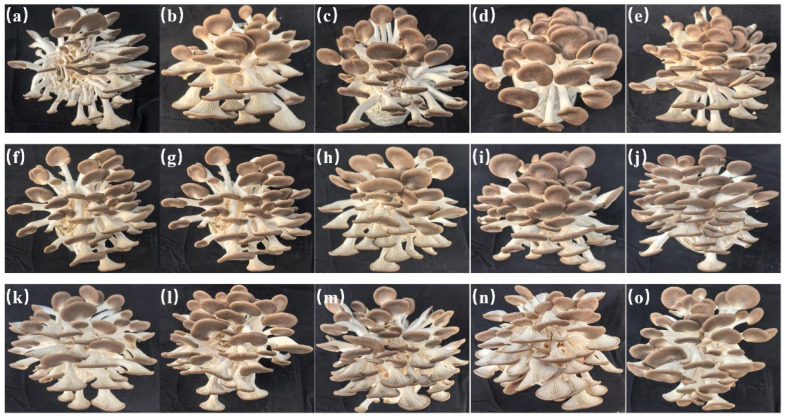
Fruiting body development of submerged cultures at different inoculation durations. (**a**): 3 d; (**b**): 3.5 d; (**c**): 4 d; (**d**): 4.25 d; (**e**): 4.5 d; (**f**): 4.75 d; (**g**): 5 d; (**h**): 5.25 d; (**i**): 5.5 d; (**j**): 5.75 d; (**k**): 6 d; (**l**): 6.25 d; (**m**): 6.5 d; (**n**): 6.75 d and (**o**): 7 d.

Table 3 and Figure 2 and Figure 3 present the effects of submerged culture inoculation time on the fruiting performance of *P. pulmonarius*. The results indicate that the duration of inoculation of submerged cultures significantly affects subsequent fruiting yield, quality and morphology of the fruiting bodies. In terms of yield and fruiting body number, bags inoculated with submerged cultures cultivated for 4.75–5.5 days achieved the best first flush yields, ranging from 281.33 g/bag to 284 g/bag. Notably, the culture incubated for 4.75 days resulted in the highest peak fruiting body number (70 bodies/bag). In terms of individual mushroom weight, mycelial cultured for 5.0 and 5.25 days produced larger fruiting bodies, with the 5.25-day treatment achieving a maximum weight of 6.39 g per mushroom. The proportion of high-quality mushrooms increased with the extension of culture duration. Strains incubated for 4.5–7.0 days maintained a high proportion of high-quality mushrooms (exceeding 83%), with the highest proportion (87.97%) observed in the 6.25-day culture. Regarding fruiting body morphology, pileus width and length remained relatively large during the 5.0–6.75-day cultivation duration. The stipe length reached its peak (46.57 mm) in the 5.0-day strain. Stipe diameter and pileus thickness exhibited relatively stable variations across cultivation durations. Furthermore, the morphology of the pileus margin progressively transitioned from irregular to smooth after 4.5 days of cultivation, indicating enhanced strain maturity.

Taking all the above results into comprehensive consideration, the optimal inoculation window for liquid spawn culture is between 4.75 and 5.5 days. During this duration, the inoculated culture exhibits multiple advantages, including high yield, a relatively high proportion of high-quality mushrooms and well-formed fruiting bodies. When culture duration exceeds 5.5 days, it is noteworthy that the proportion of high-quality mushrooms continues to increase, though yield shows a slight decline. Consequently, precise control of submerged culture inoculation duration is essential in industrialized production. Inoculation should be performed within this optimal window to simultaneously improve the yield and quality of *P. pulmonarius*.

### 3.3. Growth Curve Determination for the Formula

#### 3.3.1. Mycelial Biomass

**Figure 4:**
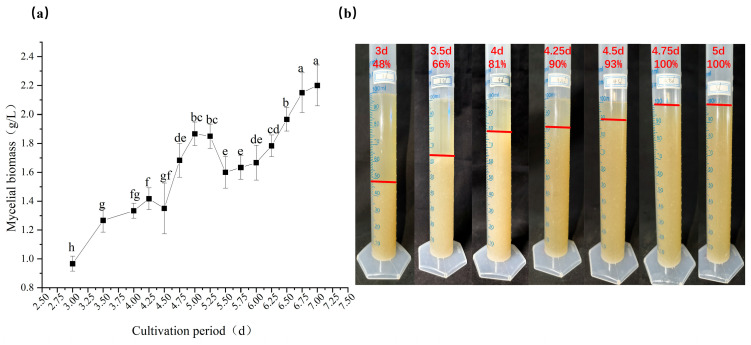
Mycelial biomass of submerged cultures at different inoculation durations. (**a**) Mycelial biomass; (**b**) Mycelial concentration. The different letters in Figure (**a**) indicate significant differences (*p* < 0.05, Duncan’s multiple range test). The red line in Figure (**b**) shows the percentage of mycelium.

Figure 4a,b illustrate the dynamic changes in mycelial biomass during the submerged culture of *P. pulmonarius*. Overall, mycelial biomass tended to increase with prolonged culture time. During the initial cultivation phase (days 3.0–5.0), mycelial biomass increased rapidly and continuously, rising sharply from 0.967 g/L on day 3.0 to 1.867 g/L on day 5.0. A particularly rapid increase occurred between days 4.25 and 5.0, during which biomass increased from 1.42 g/L to 1.87 g/L. A slight temporary decrease in mycelial biomass was observed between days 5.0 and 5.5, falling from 1.867 g/L to 1.60 g/L. Subsequently, from days 5.5 to 7.0, biomass resumed its upward trend and reached the maximum value of 2.20 g/L on day 7.0.

Preliminary fruiting experiments indicated that liquid spawn cultures exhibited optimal subsequent yield and quality when maintained for 4.75–5.5 days, corresponding to a mycelial biomass range of 1.60–1.86 g/L. This indicates that excessive mycelial biomass is not always beneficial, and moderate biomass may reflect the optimal physiological state of the strain.

#### 3.3.2. Mycelial Growth Rate on Sawdust Medium

**Figure 5:**
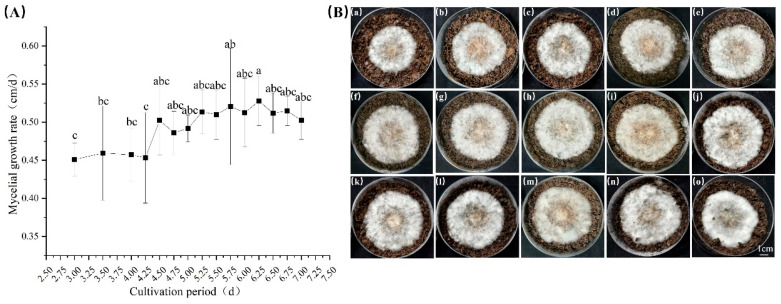
Mycelial growth of submerged cultures on sawdust medium at different inoculation durations. (**A**): Mycelial growth rate; (**B**): Mycelial growth status. (**a**): 3 d; (**b**): 3.5 d; (**c**): 4 d; (**d**): 4.25 d; (**e**): 4.5 d; (**f**): 4.75 d; (**g**): 5 d; (**h**): 5.25 d; (**i**): 5.5 d; (**j**): 5.75 d; (**k**): 6 d; (**l**): 6.25 d; (**m**): 6.5 d; (**n**): 6.75 d and (**o**): 7 d. The different letters in Figure (**A**) indicate significant differences (*p* < 0.05, Duncan’s multiple range test).

Figure 5 shows the mycelial germination and growth rates of liquid spawn cultures at different inoculation durations on a sawdust medium. As the inoculation duration of the submerged cultures increased, the mycelial growth rate on the sawdust medium exhibited an initial rise followed by a decline.

During the early culture stage (3.0–4.0 days), mycelial growth was relatively slow. From 4.5 days onward, however, the growth rate accelerated significantly and remained relatively high. The maximum mycelial growth rate on the sawdust medium was observed in the 6.25-day culture, reaching 0.53 cm/day, after which the growth rate decreased slightly.

Consistent with earlier fruiting body trials (Section 3.2), submerged cultures incubated for 4.75–5.5 days yielded the best fruiting body quality in subsequent cultivation. This phase corresponds to a duration of rapid increase in mycelial growth on sawdust medium, before the peak growth rate is reached. These results indicate that the mycelial growth rate on a sawdust medium can serve as an indicator of the vitality of liquid inoculum. However, optimal fruiting performance does not simply correlate with the maximum mycelial growth rate.

#### 3.3.3. Mycelial Pellet Diameter

**Figure 6:**
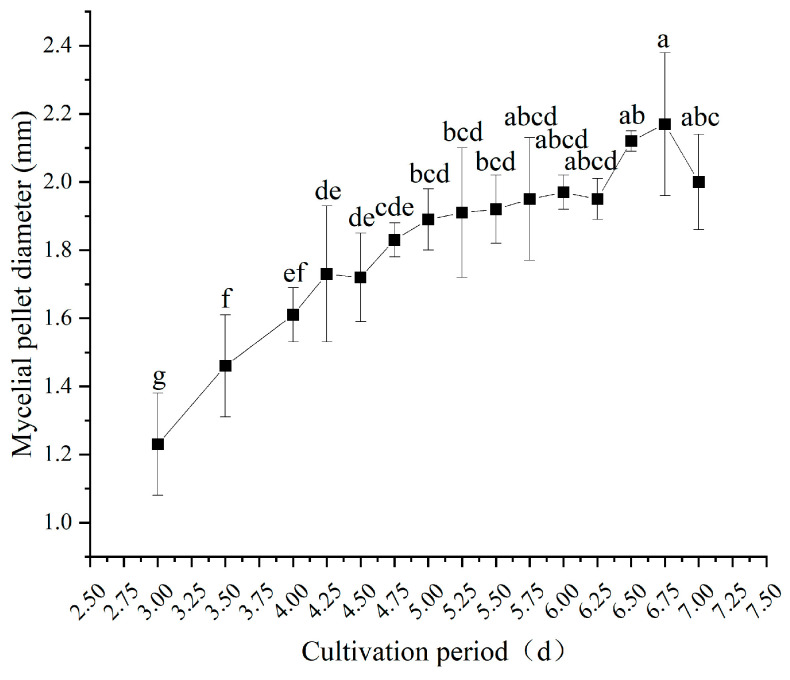
Mycelial pellet diameter in submerged cultures at different inoculation durations. Different letters indicate significant differences (*p* < 0.05, Duncan’s multiple range test).

Figure 6 illustrates the dynamic changes in mycelial pellet diameter during submerged culture. With prolonged inoculation duration, the pellet diameter exhibited sustained growth. From the early to mid-stage of cultivation (3.0 to 6.5 days), the diameter of the mycelial pellet steadily increased, rising from 1.77 mm at 3.0 days to 1.83–2.17 mm at 6.5 days. By 6.75 days, the diameter of the mycelial pellet reached its maximum value of 2.17 mm throughout the entire cultivation cycle, which was significantly higher than at other sampling time points (*p* < 0.05).

Consistent with earlier fruiting trials (Section 3.2), liquid spawn cultured between 4.75 and 5.5 days produced the best subsequent fruiting bodies in terms of both quantity and quality, with corresponding mycelial pellet diameters ranging from 1.83 mm to 1.92 mm. This suggests that excessively large or small mycelial pellet diameters can hinder optimal fruiting performance. A moderate mycelial pellet diameter (1.83–1.92 mm) can be used as a key morphological indicator for evaluating high-quality liquid spawn.

#### 3.3.4. Mycelial Pellet Density

**Figure 7:**
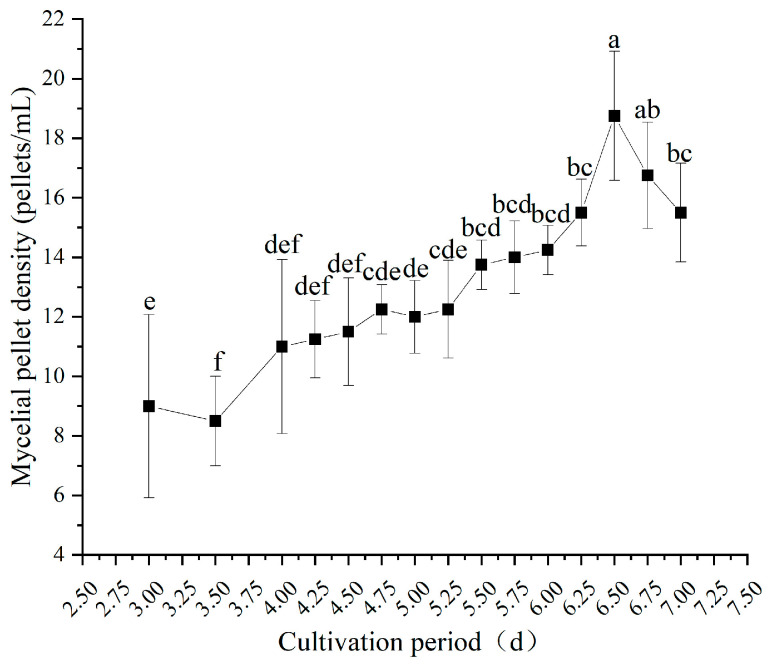
Mycelial pellet density of submerged cultures at different inoculation durations. Different letters indicate significant differences (*p* < 0.05, Duncan’s multiple range test).

Figure 7 illustrates the dynamic changes in mycelial pellet density during submerged culture. As the culture time prolonged, the mycelial pellet density initially rose rapidly, then stabilized, and finally decreased slightly. From the early to mid-cultivation phase (3.0 to 6.5 days), mycelial pellet density increased continuously. A particularly rapid increase occurred between 3.0 and 4.75 days, with density rising from 9 pellets/mL at 3.0 days to 12.25 pellets/mL at 4.75 days.

The density continued to increase and reached a peak of 18.75 pellets/mL by 6.5 days. After 6.75 days, mycelial pellet density declined slightly. Consistent with earlier fruiting trials (Section 3.2), the optimal range for subsequent fruiting yield and quality was observed in liquid spawn cultured between days 4.75 and 5.5, corresponding to a mycelial pellet density of 12.25 to 13.75 pellets/mL. This suggests that both excessively high or low mycelial pellet densities are unfavorable for ideal fruiting performance. Therefore, a moderate mycelial pellet density (12.25–13.75 pellets/mL) could be used as an indicator of high-quality submerged cultures.

#### 3.3.5. Fermentation Broth pH

**Figure 8:**
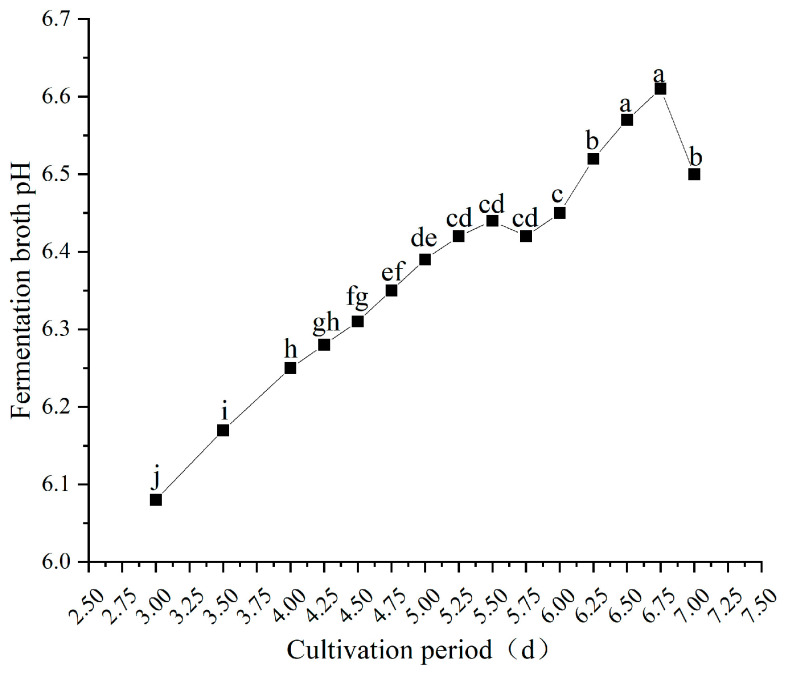
Fermentation broth pH of submerged culture at different inoculation durations. Different letters indicate significant differences (*p* < 0.05, Duncan’s multiple range test).

Figure 8 illustrates the dynamic changes in fermentation broth pH during liquid culture. As cultivation time prolonged, the pH of the fermentation broth displayed an overall trend of initially rising, followed by a subsequent decline. From the early to mid-stage of cultivation (3.0 to 6.75 days), the pH of the fermentation broth rose continuously from 6.08 at 3.0 days to 6.61 at 6.75 days, which represented the peak value for the entire cultivation cycle. Subsequently, the pH began to decline and reached 6.50 on day 7. Consistent with previous fruiting trials (Section 3.2), mushroom yield and quality were optimal when the liquid spawn was cultured between days 4.75 and 5.5 days, corresponding to a pH range of 6.35–6.44 during fermentation. This suggests that the *P. pulmonarius* culture may achieve optimal physiological metabolism within this moderate pH range, thereby supporting the formation of high-yield and high-quality fruiting bodies.

### 3.4. Correlation Analysis

**Figure 9:**
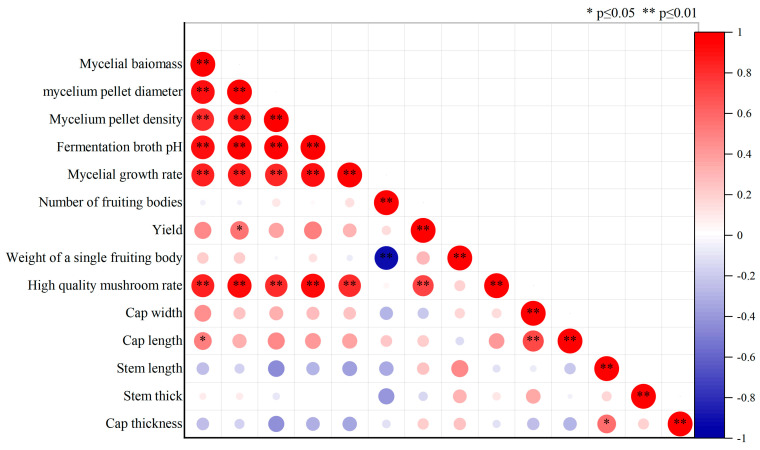
Correlation analysis of mycelial and fruiting body traits in submerged cultures at different inoculation durations.

Correlation analysis was conducted between various indicators during submerged culture and fruiting performance, with the results shown in Figure 9. The first flush yield of *P. pulmonarius* showed a highly significant positive correlation with the high-quality mushroom ratio (*r* = 0.72, *p* < 0.01) and a significant positive correlation with mycelial pellet diameter (*r* = 0.55, *p* < 0.05). The high-quality mushroom ratio showed highly significant positive correlations with mycelial biomass, mycelial pellet diameter, mycelial pellet density, fermentation broth pH and mycelial growth rate on sawdust substrate (with correlation coefficients of 0.85, 0.94, 0.82, 0.92 and 0.82 respectively, all *p* < 0.01). Furthermore, all fermentation parameters, including mycelial biomass, mycelial pellet diameter, mycelial pellet density, fermentation broth pH and mycelial growth rate on sawdust medium, were highly significantly positively correlated with each other (*p* < 0.01).

These results suggest that physicochemical parameters during submerged culture are associated with mycelial activity indicators, which affect the final mushroom yield and quality. Notably, the high-quality mushroom ratio exhibits highly significant positive correlations with multiple fermentation parameters, providing a crucial reference for evaluating the quality of liquid spawn.

## 4. Discussion

### 4.1. Nutritional Basis of Formulations

This study demonstrates that formulation 4 (0.3% soybean meal, 0.2% peptone, 0.2% corn flour, 1.6% glucose, 0.1% KH_2_PO_4_, 0.055% MgSO_4_) yielded the highest fruiting body production and comprehensive agronomic traits of *P. pulmonarius*, achieving a yield of 305.5 g per bag, which was significantly superior to other treatments. From the perspective of nutritional composition, this formulation’s advantage stems from its rational carbon-to-nitrogen structure and balanced nutrition: soybean meal and peptone, as composite organic nitrogen sources, provide amino acids and small peptides that are easily absorbed and utilized by mycelial [20,21]; corn flour functions both as a slow-release carbon source and a natural growth factor, complementing the rapid-release carbon source glucose to satisfy the nutritional demands of mycelia at different developmental stages [22]; the addition of KH_2_PO_4_ and MgSO_4_ supplements key inorganic ions such as phosphorus, potassium, and magnesium, ensuring the normal progression of basal metabolic activities [23]. This formulation establishes an optimal carbon-to-nitrogen ratio and comprehensive nutritional composition. It not only promotes the robust growth of mycelia during liquid fermentation but also, more importantly, produces highly viable liquid spawn. This facilitates rapid germination and colonization of sawdust substrates after inoculation, laying a solid foundation for high-yield and high-quality production.

To date, most studies on edible mushroom liquid spawn cultures have used a basic medium consisting of glucose + peptone/yeast extract + KH_2_PO_4_ + MgSO_4_, adopting statistical methods such as response surface modelling with mycelial biomass or polysaccharide yield as the sole optimisation objective [24,25]. This strategy is reflected in research on *Pleurotus eryngii* by Chol Jong et al. [26] and several other *Pleurotus* species [27,28], in which soluble carbon sources such as glucose, xylose and soybean extract are combined with inorganic salts to optimise the accumulation of extracellular polysaccharides or mycelial biomass. However, these studies commonly overlook the subsequent fruiting performance of the strains. In contrast, this study directly associated liquid formulation with final fruiting body yield and quality. This approach aligns more closely with the comprehensive performance criteria for ‘high-quality strains’ in industrial production, thus possessing strong practical application value. Notably, although formulation 8 produced the highest proportion of premium mushrooms (75.60%) in this study, its yield (272.5 g/bag) was lower than formulation 4’s. In contrast, formulation 4 also achieved a relatively high premium mushroom yield of 72.83%. This suggests that balancing high yield with quality is a more rational approach in industrial production [29].Compared to the single or simplified formulations widely used in previous studies, formulation contains slightly more components but achieves significant improvements in both yield and quality. The slight increase in raw material costs is fully offset by the enhanced production benefits, rendering this formulation highly valuable for large-scale industrial application where production efficiency is a core priority [30]. It should be noted that this study was limited to comparative screening based on standard formulations. Systematic experimental design methods, such as response surface methodology or orthogonal design, were not employed to analyze the interactions between medium components or to conduct in-depth optimisation. This prevented us from fully elucidating the synergistic effects between the various components. Future research could build upon these findings by adopting more systematic experimental design methods to optimise culture medium formulations further, with the aim of achieving a higher *P. pulmonarius* yield.

### 4.2. Growth Rhythm of Liquid Spawn Culture and Determination of the Optimal Inoculation Duration

This study indicates that *P. pulmonarius* liquid culture exhibits optimal culture parameters during the cultivation duration of 4.75–5.5 days. During this duration, mycelial biomass accumulates to 1.60–1.86 g/L, reaching a sufficient level without obvious senescence. The diameter of the mycelial pellet diameters at 1.83–1.92 mm with a density of 12.25–13.75 pellets/mL, which indicates that the culture medium contains abundant, uniformly dispersed active mycelial pellets. This morphology ensures sufficient germination points during inoculation, facilitating the rapid and uniform colonization of the culture bags while preventing internal hypoxia and limitations to nutrient transport caused by excessively large or dense sclerotia clusters [18]. Meanwhile, the fermentation broth maintains a stable pH plateau of 6.35–6.44, reflecting vigorous mycelial metabolism and a favorable culture environment [31].

Studies indicate that, concerning the relationship between mycelial pellet morphology and strain viability, submerged cultures with smaller mycelial pellet diameters and higher densities per unit volume exhibit faster germination rates and shorter full-bag times after inoculation. Wang Lan qing et al. [32] observed that optimal strain vitality was achieved with smaller mycelial pellet diameters (under 1.5 mm) and higher densities in shake flask cultures of *Pleurotus ostreatus* strain Tianda 300, which is largely consistent with the findings of this study. The mycelial pellet diameter (1.83–1.92 mm) and density (12.25–13.75 pellets/mL) within the optimal inoculation window fall precisely within a moderate range rather than an extreme range. This suggests that strain vitality does not exhibit a simple linear relationship with mycelial pellet size or quantity, but exists within an optimal range.

With regard to pH changes in the fermentation broth, Yu Changxia et al. [33] observed an initial decrease in the pH of *Pleurotus ostreatus* submerged culture before it stabilized during cultivation. This differs from the ‘increase first and then decrease’ in the present study, which may be due tothe distinct metabolic characteristics of different fungal strains. As a thermophilic fungus, *P. ostreatus* exhibits a strong capacity for acid production [34]. In contrast, when *P. pulmonarius* mycelial utilizes organic nitrogen sources such as peptone or soybean meal, deamination reactions occur, releasing alkaline substances such as ammonia. This process leads to an initial increase in pH. The temporary decline in mycelial biomass observed in this study between 5.0 and 5.5 days of cultivation, similar to that observed in submerged cultures of *Pleurotus ostreatus*, may be related to the transition from primary metabolism to secondary metabolism during this phase, as well as the partial autolysis of mycelia [32].

Compared with studies on other mushroom liquid cultures, this research employed more refined monitoring of growth rhythm (with 15 sampling time points) and conducted correlation analysis between fermentation parameters and final fruiting body traits, which has rarely been reported in existing literature [33]. The correlation analysis revealed highly significant positive correlations between the proportion of high-quality mushrooms and mycelial biomass, mycelial pellet diameter, mycelial pellet density, fermentation broth pH and mycelial germination rate (*r* > 0.8, *p* < 0.01). This provides a statistical foundation for establishing a multi-parameter quality standard.

### 4.3. Industrial Significance of Establishing Multi-Parameter Quality Standards

The multi-parameter composite quality standard proposed in this study, centred on mycelial biomass, mycelial pellet diameter, mycelial pellet density, and fermentation broth pH, represents a significant breakthrough from traditional single-indicator evaluation systems. Correlation analysis revealed highly significant positive correlations between high-quality mushroom ratio and mycelial biomass, mycelial pellet diameter, mycelial pellet density, fermentation broth pH, and mycelial growth rate on sawdust substrate (*r* = 0.82–0.94, *p* < 0.01). This indicates that these indicators reflect the quality characteristics of the fungal strain from different dimensions, showing complementarity and irreplaceability. All these indicators can be quantitatively determined using conventional equipment such as microscopes, weighing scales, and pH meters, thereby eliminating the subjectivity of empirical judgements. This approach is more comprehensive and scientific compared to the “single biomass indicator” method [35,36] widely adopted in current liquid spawn research. Most indicators (e.g., pH, mycelial pellet morphology) can be monitored in real-time or periodically during the fermentation process, facilitating timely adjustments to process parameters or determination of production termination points in industrial practice. For instance, when the fermentation broth pH reaches 6.35–6.44 and mycelial pellet diameter falls within 1.83 mm–1.92 mm, the strain is deemed optimally ready for inoculation. This approach is similar to the “84–96 h inoculation endpoint” proposed in straw mushroom submerged culture studies [33], but this research provides more specific multi-parameter criteria. The core innovation of this study is that all the established parameter ranges are directly correlated with the final fruiting yield and high-quality mushroom ratio, rather than merely focusing on the mycelial growth phase. This makes the proposed standard not only applicable for evaluating the “mycelial growth quality” but also for predicting the “fruiting quality”, thereby enhancing its practical guiding value in industrial production. In contrast, most existing studies have only focused on optimizing mycelial biomass or mycelial pellet morphology [37,38], failing to establish quantitative links between intermediate indicators and final yield.

Implementing this standard in production practice resolves common challenges in industrial cultivation, such as uneven fruiting, inconsistent yields and deteriorating quality, which are often caused by variations in spawn quality. Research indicates that achieving efficient industrial production requires consistent batch-to-batch stability of liquid spawn [39]. The multi-parameter quality standards established in this study provide a feasible solution for quality control at critical points during batch production in large fermentation tanks, ensuring that every batch of spawn leaving the facility is of optimal vitality. This is key to achieving year-round, standardised and highly efficient production of *P. pulmonarius*. However, the multi-parameter quality criteria proposed in this study are based on preliminary results obtained under specific strain and experimental conditions. To validate their broad applicability, independent research must be conducted at different production scales and in different geographical locations, using different strains.

## 5. Conclusions

This study aims to provide reference parameters for quality control of liquid spawn in the industrial production of *P. pulmonarius*. Systematic screening revealed that the optimal medium formulation was as follows: glucose (1.6%), soybean meal (0.3%), corn flour (0.2%), peptone (0.2%), KH_2_PO_4_ (0.1%) and MgSO_4_ (0.055%). Growth rhythm analysis based on this formulation indicates that the optimal cultivation and inoculation window for liquid spawn is 4.75–5.5 days. During this duration, mycelial biomass ranges from 1.60 to 1.86 g/L, with mycelial pellet diameter ranging from 1.83 to 1.92 mm, mycelial pellet density 12.25 to 13.75 pellets/mL, and fermentation broth pH 6.35 to 6.44. These four indicators collectively form a quantifiable and operable composite quality standard. Real-time monitoring of the liquid spawn in the fermentation tank enables precise determination of inoculation timing. This ensures high batch consistency and strong spawn vitality, thereby providing a solid foundation for the stable and high-quality cultivation of *P. pulmonarius*.

## Figures and Tables

**Table 1:** Submerged culture formula.

Formula	Soybean Meal (%)	Peptone (%)	Glucose (%)	Corn Flour (%)	KH_2_PO_4_ (%)	MgSO_4_ (%)
1	0.3	0.2	1.6	0.2	-	-
2	0.3	0.2	1.6	0.2	0.1	-
3	0.3	-	1.6	-	0.1	0.055
4	0.3	0.2	1.6	0.2	0.1	0.055
5	0.3	0.2	1.6	0.2	-	0.055
6	0.4	0.2	1.6	0.2	-	-
7	0.4	0.2	1.6	-	0.1	0.055
8	0.4	0.2	1.6	0.2	0.1	0.055
9	0.4	-	1.6	0.2	0.1	0.055
10	0.4	-	1.6	-	0.1	0.055

Note: - indicates no addition.

**Table 2:** Growth of *P. pulmonarius* fruiting bodies under different submerged culture formulations.

Formula	Yield (g/bag)	High Quality Mushroom Rate (%)	L	Pileus Width (mm)	Pileus Length (mm)	Stipe Length (mm)	Stipe Diameter (mm)	Pileus Thickness (mm)
1	278.0 ± 2.25 b	63.49 ± 1.00 d	38.68 ± 3.8 abc	45.62 ± 0.52 a	38.10 ± 1.20 ef	42.07 ± 1.79 c	8.87 ± 0.55 b	11.95 ± 0.36 a
2	275.0 ± 1.31 c	58.00 ± 0.92 f	36.85 ± 3.6 bcd	44.17 ± 1.91 d	38.80 ± 2.80 de	45.17 ± 2.74 ab	9.24 ± 0.58 a	11.31 ± 0.78 b
3	252.0 ± 1.99 g	71.83 ± 1.22 b	36.65 ± 2.91 cd	47.03 ± 1.82 d	39.94 ± 2.07 bcd	41.18 ± 0.97 c	8.58 ± 0.51 c	10.97 ± 0.57 bc
4	305.5 ± 2.26 a	72.83 ± 0.89 b	39.45 ± 3.61 abc	48.99 ± 3.74 c	42.56 ± 4.90 a	45.79 ± 1.85 a	9.30 ± 1.26 a	10.99 ± 0.56 bc
5	242.5 ± 2.13 h	74.64 ± 0.99 a	40.15 ± 3.61 ab	45.20 ± 4.01 bc	40.16 ± 0.91 bcd	45.21 ± 2.73 ab	7.87 ± 0.59 f	10.80 ± 0.40 bc
6	263.5 ± 2.6 e	64.14 ± 0.58 d	37.4 ± 2.47 bcd	48.36 ± 2.08 d	40.86 ± 2.36 bc	39.50 ± 0.52 d	7.78 ± 0.60 f	10.49 ± 0.55 cd
7	260.5 ± 2.64 f	61.23 ± 1.14 e	39.33 ± 4.92 abc	44.71 ± 2.21 c	40.06 ± 2.97 bcd	36.00 ± 1.23 e	8.32 ± 0.61 d	10.00 ± 1.13 d
8	272.5 ± 1.26 d	75.60 ± 1.46 a	41.03 ± 2.66 a	48.40 ± 2.40 b	41.45 ± 1.64 ab	38.15 ± 1.44 d	8.11 ± 0.70 e	9.41 ± 0.68 e
9	207.0 ± 2.67 i	42.51 ± 1.87 g	38.75 ± 4.02 abc	47.03 ± 2.27 c	39.72 ± 1.27 cd	38.38 ± 1.74 d	8.74 ± 0.61 bc	11.18 ± 1.68 b
10	176.5 ± 1.89 g	66.29 ± 1.54 c	34.8 ± 2.33 d	44.17 ± 2.08 e	36.67 ± 2.37 vef	43.87 ± 2.97 b	8.09 ± 0.77 e	10.06 ± 0.45 d

Note: Different letters indicate significant differences (*p* < 0.05, Duncan’s multiple range test).

**Table 3:** Fruiting body production of submerged cultures at different inoculation durations.

Inoculation Duration (d)	Number of Fruiting Bodies (bodies/bag)	Yield (g/bag)	Weight of a Single Fruiting Body (g)	High Quality Mushroom Rate (%)	Pileus Width (mm)	Pileus Length (mm)	Stipe Length (mm)	Stipe Diameter (mm)	Pileus Thickness (mm)	Pileus Margin Morphology
3.0	62 ± 43.31 c	244.00 ± 3.86 f	3.94 ± 0.11 i	75.82 ± 1.38 g	47.47 ± 3.48 bcde	39.12 ± 3.5 abc	42.25 ± 3.11 abc	11.15 ± 1.14 c	9.41 ± 1.96 f	irregular
3.5	66 ± 1.79 b	233.33 ± 2.67 g	3.54 ± 0.06 g	77.43 ± 1.38 f	46.70 ± 4.43 cde	38.16 ± 3.12 abc	43.12 ± 4.39 abc	11.51 ± 0.65 bc	11.76 ± 0.90 abcd	irregular
4.0	43 ± 2.02 g	245.33 ± 2.54 f	5.71 ± 0.13 c	78.35 ± 1.06 f	47.27 ± 3.49 bcde	38.88 ± 2.22 c	43.89 ± 3.88 abc	11.29 ± 0.66 c	12.18 ± 0.83 abc	irregular
4.25	46 ± 1.5 f	229.33 ± 1.68 h	4.99 ± 0.07 d	80.52 ± 0.72 e	50.85 ± 3.73 abc	37.66 ± 2.28 abc	45.26 ± 3.56 abc	12.69 ± 1.24 a	11.21 ± 0.83 bcde	irregular
4.5	62 ± 1.97 c	269.33 ± 1.91 b	4.34 ± 0.15 g	83.67 ± 1.65 d	46.12 ± 2.08 de	37.52 ± 1.99 bc	43.12 ± 4.57 abc	11.99 ± 0.39 abc	12.78 ± 1.34 a	smooth
4.75	70 ± 1.57 a	284.00 ± 1.96 a	4.06 ± 0.07 h	85.63 ± 1.03 c	45.30 ± 2.46 e	38.60 ± 3.04 abc	43.60 ± 6.09 abc	11.40 ± 0.74 c	12.80 ± 0.91 a	smooth
5.0	46 ± 1.76 f	282.33 ± 1.67 a	6.14 ± 0.07 b	86.26 ± 1.17 bc	48.51 ± 2.1 ab	40.53 ± 3.52 ab	46.57 ± 4.36 a	12.61 ± 083 ab	12.42 ± 1.82 ab	smooth
5.25	44 ± 1.52 fg	281.33 ± 1.04 a	6.39 ± 0.09 a	86.69 ± 1.32 abc	47.74 ± 2.78 bcde	39.33 ± 2.23 abc	44.41 ± 4.99 abc	11.47 ± 0.49 c	10.89 ± 0.89 cdef	smooth
5.5	68 ± 1.59 b	283.00 ± 1.33 a	4.16 ± 0.05 h	86.43 ± 1.10 abc	46.69 ± 2.55 cde	38.64 ± 1.97 abc	46.12 ± 4.52 ab	11.13 ± 1.16 c	10.74 ± 0.99 cdef	smooth
5.75	53 ± 2.2 e	264.00 ± 2.05 c	4.98 ± 0.06 d	87.36 ± 1.82 ab	46.69 ± 4.84 cde	38.37 ± 3.81 abc	44.44 ± 3.83 abc	11.54 ± 1.2 bc	11.73 ± 1.42 abcd	smooth
6.0	55 ± 1.85 e	265.33 ± 2.49 c	4.82 ± 0.05 e	86.53 ± 1.5 abc	43.83 ± 3.21 e	35.20 ± 1.99 c	41.34 ± 3.84 abc	12.24 ± 1.3 abc	10.14 ± 1.58 ef	smooth
6.25	60 ± 3.33 cd	261.33 ± 3.53 d	4.36 ± 0.18 fg	87.97 ± 1.66 a	49.81 ± 5.47 abcd	40.54 ± 2.6 ab	41.62 ± 4.09 abc	12.09 ± 1.00 abc	10.30 ± 1.38 def	smooth
6.5	61 ± 3.8 cd	264.00 ± 3.82 c	4.33 ± 0.15 g	87.53 ± 1.68 ab	50.91 ± 4.04 abc	42.41 ± 3.56 a	40.77 ± 4.05 bc	11.26 ± 1.10 c	10.31 ± 1.26 def	smooth
6.75	60 ± 2.33 cd	268.00 ± 3 b	4.47 ± 0.16 f	87.55 ± 1.77 ab	48.11 ± 3.33 bcde	38.70 ± 2.38 abc	42.13 ± 4.13 abc	11.08 ± 1.16 c	9.92 ± 1.16 ef	smooth
7.0	59 ± 2.39 d	252.00 ± 1.76 e	4.27 ± 0.11 g	86.67 ± 1.4 abc	52.68 ± 4.27 a	41.09 ± 3.60 ab	40.27 ± 5.87 c	11.99 ± 1.05 abc	9.74 ± 1.46 ef	smooth

Note: Different letters indicate significant differences (*p* < 0.05, Duncan’s multiple range test).

## Data Availability

Data will be made available on request.
